# Comprehensive Mapping of Common Immunodominant Epitopes in the Eastern Equine Encephalitis Virus E2 Protein Recognized by Avian Antibody Responses

**DOI:** 10.1371/journal.pone.0069349

**Published:** 2013-07-26

**Authors:** EnCheng Sun, Jing Zhao, Liang Sun, QingYuan Xu, Tao Yang, YongLi Qin, WenShi Wang, Peng Wei, Jing Sun, DongLai Wu

**Affiliations:** The Key Laboratory of Veterinary Public Health, Ministry of Agriculture, State Key Laboratory of Veterinary Biotechnology, Harbin Veterinary Research Institute, Chinese Academy of Agricultural Sciences, Harbin, People's Republic of China; Northeast Agricultural University, China

## Abstract

Eastern equine encephalitis virus (EEEV) is a mosquito-borne virus that can cause both human and equine encephalitis with high case fatality rates. EEEV can also be widespread among birds, including pheasants, ostriches, emu, turkeys, whooping cranes and chickens. The E2 protein of EEEV and other *Alphaviruses* is an important immunogenic protein that elicits antibodies of diagnostic value. While many therapeutic and diagnostic applications of E2 protein-specific antibodies have been reported, the specific epitopes on E2 protein recognized by the antibody responses of different susceptible hosts, including avian species, remain poorly defined. In the present study, the avian E2-reactive polyclonal antibody (PAb) response was mapped to linear peptide epitopes using PAbs elicited in chickens and ducks following immunization with recombinant EEEV E2 protein and a series of 42 partially overlapping peptides covering the entire EEEV E2 protein. We identified 12 and 13 peptides recognized by the chicken and duck PAb response, respectively. Six of these linear peptides were commonly recognized by PAbs elicited in both avian species. Among them five epitopes recognized by both avian, the epitopes located at amino acids 211–226 and 331–352 were conserved among the EEEV antigenic complex, but not other associated alphaviruses, whereas the epitopes at amino acids 11–26, 30–45 and 151–166 were specific to EEEV subtype I. The five common peptide epitopes were not recognized by avian PAbs against Avian Influenza Virus (AIV) and Duck Plague Virus (DPV). The identification and characterization of EEEV E2 antibody epitopes may be aid the development of diagnostic tools and facilitate the design of epitope-based vaccines for EEEV. These results also offer information with which to study the structure of EEEV E2 protein.

## Introduction

Eastern equine encephalitis virus (EEEV) is an arbovirus that causes severe neurological disease in humans and equines throughout the Americas [1]. EEEV is recognized as a potential agent of biowarfare and bioterrorism, and is listed as a National Institute of Allergy and Infectious Disease (NIAID) Category B priority pathogen and as a Human Health and Services (HHS) select agent [2]. EEEV belongs to the family *Togaviridae*, genus *Alphavirus*, and is considered a New World Alphavirus along with Venezuelan equine encephalitis Virus (VEEV) and Western equine encephalitis Virus (WEEV), as opposed to the Old World Alphaviruses, which include the Ross River virus (RRV), Semliki Forest virus (SFV) and Sindbis virus (SINV) [3]. On the basis of the difference of hemagglutination inhibition activity, EEEV can be divided into two types, North America (NA) and South America (SA). NA EEEV is represented by only one subtype, subtype I, which is highly conserved across various geographic locations and over time. In contrast, SA EEEV includes subtypes II–IV, which are associated with different geographic regions [4]–[6].

NA EEEV is transmitted among migratory passerine songbirds, starlings, and wading birds in freshwater swamps by mosquitoes [7], and is also an important pathogen of mammalian hosts, including equids and humans. In addition to playing important roles in expanding geographical distribution of EEEV, bird hosts are also necessary for the amplification of NA EEEV [8]. High attack and fatality rates are commonly associated with EEEV infection of horses, but has also been documented in swine [9], pheasants [10], ostriches [11], emus [12], turkeys [13] and whooping cranes [14]. Many domesticated birds can be infected with EEEV by pecking and preening [15], and subsequently develop both a viscerotropic disease and encephalitis after EEEV infection [16]. Usually several weeks before EEEV becomes enzootic, EEEV-reactive antibodies can be detected in samples collected from local birds and virus can be isolated. Thus, avian species and specifically chickens can serve as sentinels to monitor EEEV activity. SA EEEV is principally an equine pathogen with high susceptibility and fatality rates, but SA EEEV infections are rarely detected in humans. Even during major equine epizootics, only three human cases have been described [17]. Horse epizootics have occurred in Panama [18], Brazil [19] and Argentina [20], and hamsters can act as a sentinel species for EEEV in the Catatumbo region [21]. However, up to now, there is no definitive information on the reservoir and amplification hosts of SA EEEV. Studies evaluating EEEV seroprevalence and experimental infection parameters suggest that rodents may play an important role in tropical EEEV transmission [1].

The genome of EEEV is a single-stranded, positive-sense RNA of approximately 11.7 kb that is capped at the 5′ end and polyadenylated at the 3′ end. The EEEV genome encodes two open reading frames (ORFs) for the non-structural and structural polyproteins. The nonstructural proteins (NSP1-4) are involved with the transcription and replication of viral RNA, polyprotein cleavage, and RNA capping during the virus replication process [22]. The structural proteins include the capsid protein, and envelope glycoproteins E3, E2, 6K and E1. The EEEV structural proteins are involved in receptor recognition, virus attachment and penetration, membrane fusion, virion assembly, as well as other viral functions [23]. Among structural proteins, the E2 protein is highly immunogenic in the context of infection and immunization, and elicits neutralizing and hemagglutination inhibiting antibodies. E2 protein-reactive antibodies are known to limit EEEV infection and inhibit viral RNA levels in infected cells [2], [23].

As a virally encoded virion glycoprotein, the E2 proteins of alphaviruses have important antigenic characteristics of diagnostic value. Administration of VEEV E2-specific monoclonal antibodies (MAbs) provided broad protection against several different serogroups in murine models with a protection rate over 75% [24]. The EEEV E2 protein-specific MAbs have been used in the EEEV antigen-capture ELISA assays for infected mosquito surveillance [25]. Identification of E2 protein-specific antibody epitopes will further contribute to the development of diagnostic tests based on EEEV E2 protein antigenicity. Most information regarding antibody recognition of EEEV E2 stems from studies of the murine immune response [26], and there have been no reports describing E2 epitopes recognized by antibodies generated in avian species. In this study, we characterized the E2 protein epitopes recognized by the avian antibody response, and defined the common immunodominant E2 epitopes that were targeted by antibodies in chickens and ducks. Furthermore, we evaluate the conservation of the commonly recognized epitopes among the EEEV lineages and associated alphaviruses, and defined epitopes as EEEV lineage I-specific or EEEV antigenic complex-specific. These results provide a foundation for the development of diagnostic assays for the different EEEV lineages and will facilitate the design of epitope-based EEEV vaccines. Moreover, the resolution of antibody binding E2 epitopes can be applied to studies to define the reservoirs and amplification hosts for NA and SA EEEV.

## Results

### E2-reactive avian PAb titers

Chickens and ducks were immunized with purified recombinant E2 protein to elicit E2-reactive antibodies. Antibody titers were determined by indirect ELISA and an immunofluorescence assay (IFA) using sera collected prior to each immunization and serum from the terminal blood collection which was performed two weeks after the final booster immunization. The titer of EEEV E2-reactive antibodies increased with each sequential immunization of chickens and ducks, whereas unimmunized control animals did not have detectable levels of E2 protein-reactive antibodies at any time point (Table 1). The final PAb titers from chicken and duck were 1∶10^6^ and 1∶10^5^, respectively, when measured using an indirect ELISA. By IFA using E2 expressed in BHK-21 cells, E2-reactive PAb titers were 1∶256 for the chicken sera and 1∶128 for the duck sera.

**Table 1:** Determination of the titers of PAbs from different species by IFA/ELISA.

	Immunization Time Points				
Titer of PAbs	0 week	2nd week	4th week	5th week	6th week
Chicken PAbs	−/−	1∶32/1∶10^3^	1∶64/1∶10^4^	1∶256/1∶10^6^	1∶256/1∶10^6^
Chicken control	−/−	−/−	−/−	−/−	−/−
Duck PAbs	−/−	1∶16/1∶10^2^	1∶64/1∶10^4^	1∶128/1∶10^5^	1∶128/1∶10^5^
Duck control	−/−	−/−	−/−	−/−	−/−

−, titer below the limit of detection (LOD): IFA = 1∶2; LOD ELISA = 1∶10.

Left showed the value detected by IFA, and right showed the value detected by ELISA.

### Comprehensive mapping of linear avian epitopes on EEEV E2 protein

We next sought to identify antibody binding linear epitopes on the EEEV E2 protein. The PAbs elicited in chickens and ducks through immunization with recombinant E2 protein were used to screen a series of 42 partially overlapping peptides derived from the entire EEEV E2 protein by Western blot (WB). Each peptide was 16 amino acids in length and was expressed as a fusion with mannose binding protein (MBP). As shown in Table 2, 12 peptides in the series were recognized by chicken PAbs (E2-2/4/15/16/18/19/22/24/30/31/34/35). Thirteen peptides were recognized by duck PAbs (E2-1/2/3/4/5/6/11/12/16/22/32/34/35). Six peptide epitopes were recognized by both PAbs in the serum of immunized chickens and ducks (E2-2/4/16/22/34/35). There were six other E2 peptide epitopes specifically recognized by the chicken antibody response that were not recognized by the PAbs elicited in duck, and seven E2 peptide epitopes recognized by the duck antibody response that were not recognized by the PAbs elicited in chicken. As expected, PAbs in the serum of ducks and chickens recognized the full-length purified E2 protein, and no reactivity of antisera with the MBP-tag alone was observed. Sera from unimmunized poultry did not react with any of the 42 MBP-fused polypeptides or MBP-tag alone (data not shown).

**Table 2:** Identification of linear peptide epitopes in the EEEV E2 protein using PAbs from avian by WB.

PAbs Origin	Peptides Denomination	E2-1	E2-2	E2-3	E2-4	E2-5	E2-6	E2-7	E2-8	E2-9	E2-10	E2-11	E2-12	E2-13	E2-14	E2-15	E2-16	E2-17	E2-18	E2-19	E2-20	E2-21	E2-22
Avian	Chicken		+		+											+	+		+	+			+
	Duck	+	+	+	+	+	+					+	+				+						+

PAbs Origin	Peptides Denomination	E2-23	E2-24	E2-25	E2-26	E2-27	E2-28	E2-29	E2-30	E2-31	E2-32	E2-33	E2-34	E2-35	E2-36	E2-37	E2-38	E2-39	E2-40	E2-41	E2-42	E2 protein	MBP-tag
Avian	Chicken		+						+	+			+	+								+	
	Duck										+		+	+								+	

**Note: +** represents positive reaction with respectively expressed polypeptide using antisera by WB.

### Confirmation of the PAb epitopes on the E2 protein by peptide ELISA

**Figure 1:**
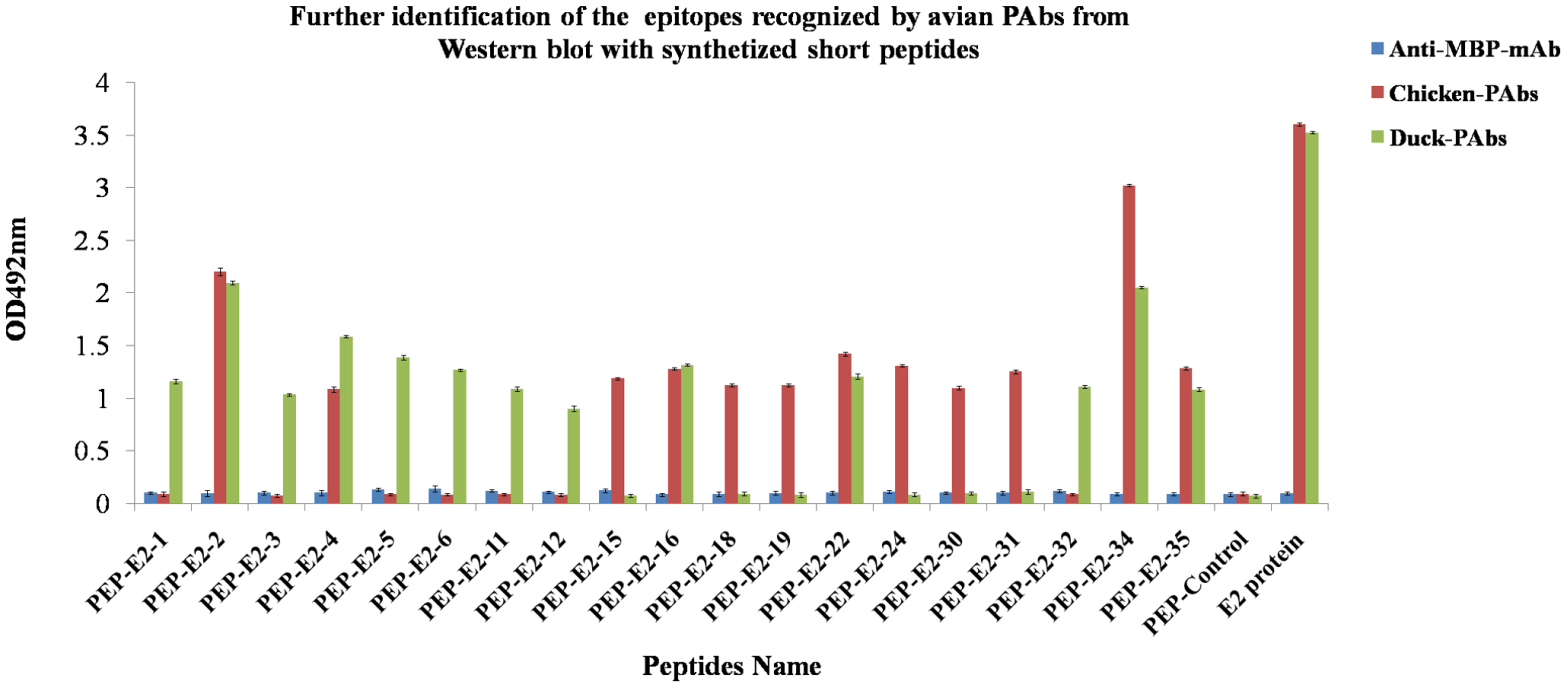
Verification of the E2 peptide epitopes recognized by PAbs raised in different avian. E2-reactive PAbs were elicited in chickens and ducks immunized with purified recombinant EEEV E2 protein. A Western blot screen of forty-two 16-mer peptides derived from the E2 protein sequence identified a panel of putative peptide epitopes recognized by the E2-reactive PAbs of one or more species. The candidate epitopes were synthesized as short peptides and used in an ELISA to measure PAb binding to each linear peptide epitope. PAb binding was determined by measuring optical density (OD) at 492 nm. Error bars indicate standard deviation. Positive value/Negative value ≥2.1 was considered as positive result.

We next confirmed the reactivity of avian E2-reactive PAbs with the identified peptide epitopes. The 19 candidate polypeptides identified in the WB screen were synthesized and screened by peptide ELISA using the poultry PAbs. The pattern of PAb binding to the 19 candidate peptides by ELISA was consistent with the results of the WB screen against the E2 peptide series expressed as MBP fusion proteins. The six E2 epitopes commonly recognized by chicken and duck PAb responses by WB (E2-2/4/16/22/34/35) were also recognized by the indicated PAb response when the peptides were synthesized and used as target antigen in an ELISA (Figure 1). As expected, PAbs in the serum of ducks and chickens recognized the full-length purified E2 protein in the ELISA, whereas there was no reactivity of the antisera with an irrelevant peptide control and the anti-MBP-mAb (antibody control) did not react with any polypeptides and E2 protein (Figure 1). Sera from unimmunized poultry did not react with any of the synthesized polypeptides and E2 protein (data not shown).

### Location analysis of the epitopes in the E2 protein

**Figure 2:**
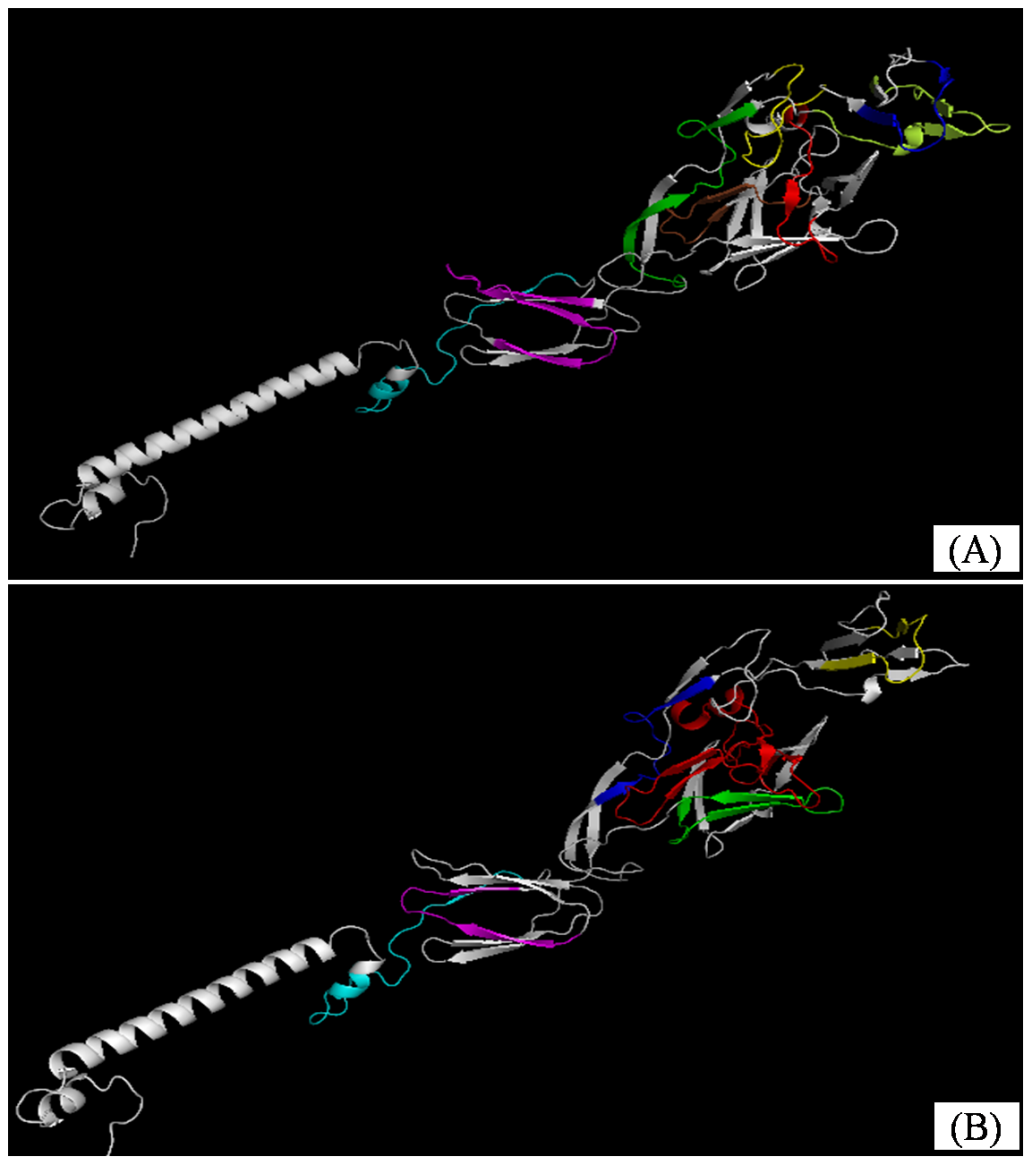
Location analysis of the epitopes in the EEEV E2 protein. A and B showed ribbon diagram of the epitopes recognized by chicken and duck immune response on EEEV E2 protein, respectively. The epitope E2-2 (11-26aa), 4 (31-46aa), 15+16 (141-166aa), 18+19 (171-196aa), 22 (211-226aa), 24 (231–246), 30+31 (291–316) and 34+35 (331–356) in figure A was showed by color red, brown, green, limen, blue, yellow, magenta and cyan, respectively. And the epitope E2-1∼6 (2-66aa), 11/12 (101-126aa), 16 (151-166aa), 22 (211-226aa), 32 (311–326) and 34+35 (331–356) in figure B was showed by color red, green, blue, yellow, magenta and cyan, respectively.

PEPSCAN analysis of PAbs from immunized chicken and duck identified 16-residue peptides of the EEEV E2 protein that were targeted by the poultry immune system. For comprehensive analysis spatial distribution of the identified epitopes on E2 protein, homology modeling and structural visualization were employed to identify the specific series residues in each epitope responsible for antibodies. Finally, the locations of the epitopes on the surface of the E2 homology model were visualized (Figure 2).

### Analysis of the conservation of the five common epitopes among alphaviruses

**Figure 3:**
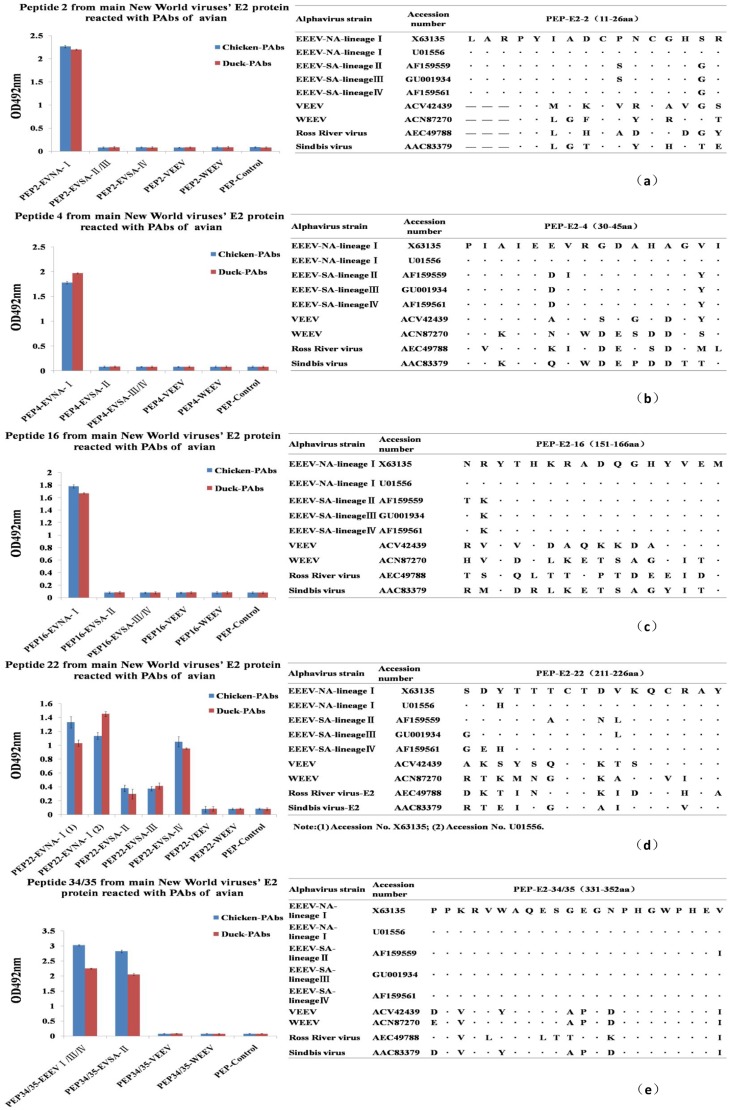
Specificity of avian-specific E2 peptide epitopes among associated alphaviruses. Amino acid alignments of the three commonly recognized EEEV E2 peptide epitopes with the corresponding region of E2 from other EEEV lineages and associated alphaviruses were performed (right panels). The corresponding peptides were synthesized and used as target antigen in ELISA to determine if the chicken and duck PAbs were specific for individual EEEV lineages, the entire EEEV antigen complex, or also recognized VEEV and WEEV (left panels). Positive value/Negative value ≥2.1 was considered as positive result. The sequences from Ross River Virus and Sindbis Virus are shown for comparison.

We next evaluated the conservation of the five common EEEV E2 linear peptide epitopes recognized by avian PAbs among EEEV antigen complex viruses and other associated alphaviruses. The regions corresponding to the five common EEEV E2 epitopes were identified by aligning amino acid sequences of EEEV antigen complex viruses and other associated alphaviruses (Figure 3). When the amino acid sequence in the identified epitope region differed by one or more amino acids from the EEEV NA lineage I E2 epitope sequence, the peptide was synthesized and tested by ELISA using chicken and duck EEEV E2 antisera. We found that the E2-2, 4 and 16 peptide epitopes, located at amino acids 11–26, 30–45 and 151–166, were EEEV lineage I-specific epitopes, as peptides synthesized from the corresponding region of EEEV lineages II–IV, VEEV and WEEV were not recognized by chicken or duck antisera (left panels of Figure 3 a, b and c). The E2-22 and 34/35 epitope, located at amino acids 211–226 and 331–352 of the E2 protein, was conserved among all EEEV lineages, but peptides synthesized based on the corresponding region of VEEV and WEEV were not recognized by the chicken and duck antisera (Figure 3 d and e). As expected, no reactivity was detected between the PAbs and an irrelevant peptide control. Similarly, the sera from unimmunized poultry did not detectably react with any of the synthesized peptides (data not shown).

**Figure 4:**
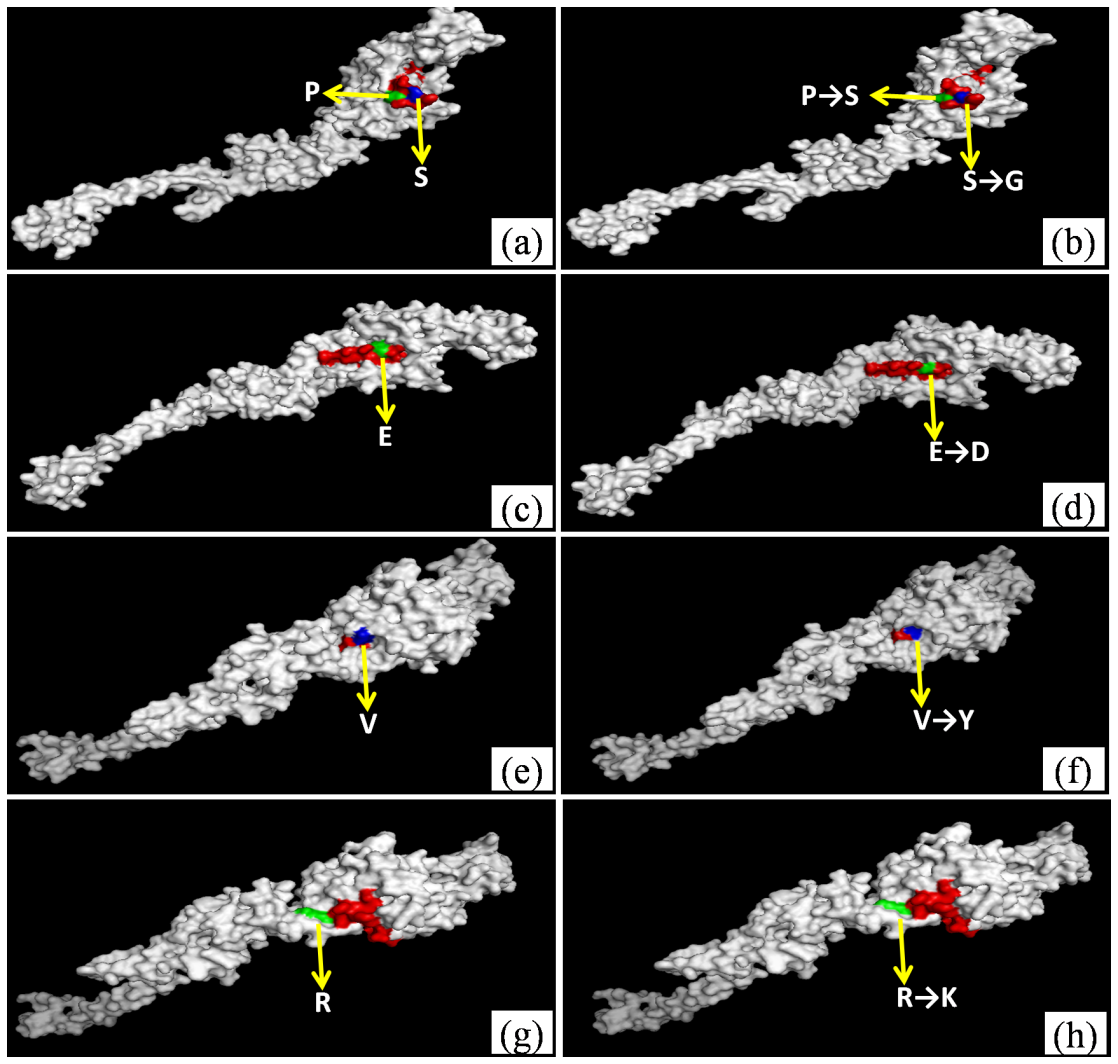
Analysis of conformation change of amino acid substitution in the EEEV subtype I epitopes. Comprehensively analysis the amino acid residue substitution using 3D structure showed that if amino acid substitution would influence on the structure of EEEV E2 protein. a and b: ^20^P→S^20^ and ^25^S→G^25^ mutation; c and d: ^35^E→D^55^ mutation; e and f: ^44^V→Y^44^ mutation; g and h: ^152^R→K^152^ mutation.

Then we used the software to analyze of the amino acid substitution in EEEV type-I epitopes to determine if the substitution would influence on the conformation of E2 protein (Figure 4). The results showed: 20P→S20 and 25S→G25 (Figure 4 a and b), 35E→D55 (Figure 4 c and d), 44V→Y44 (Figure 4 e and f) and 152R→K152 (Figure 4 g and h) did not influence on protein conformation, but after 44V→Y44 mutation (Figure 4 e and f) the amino acid showed little invagination.

### Antisera elicited by AIV and DPV do not recognize the five common immunodominant E2 epitopes

**Figure 5:**
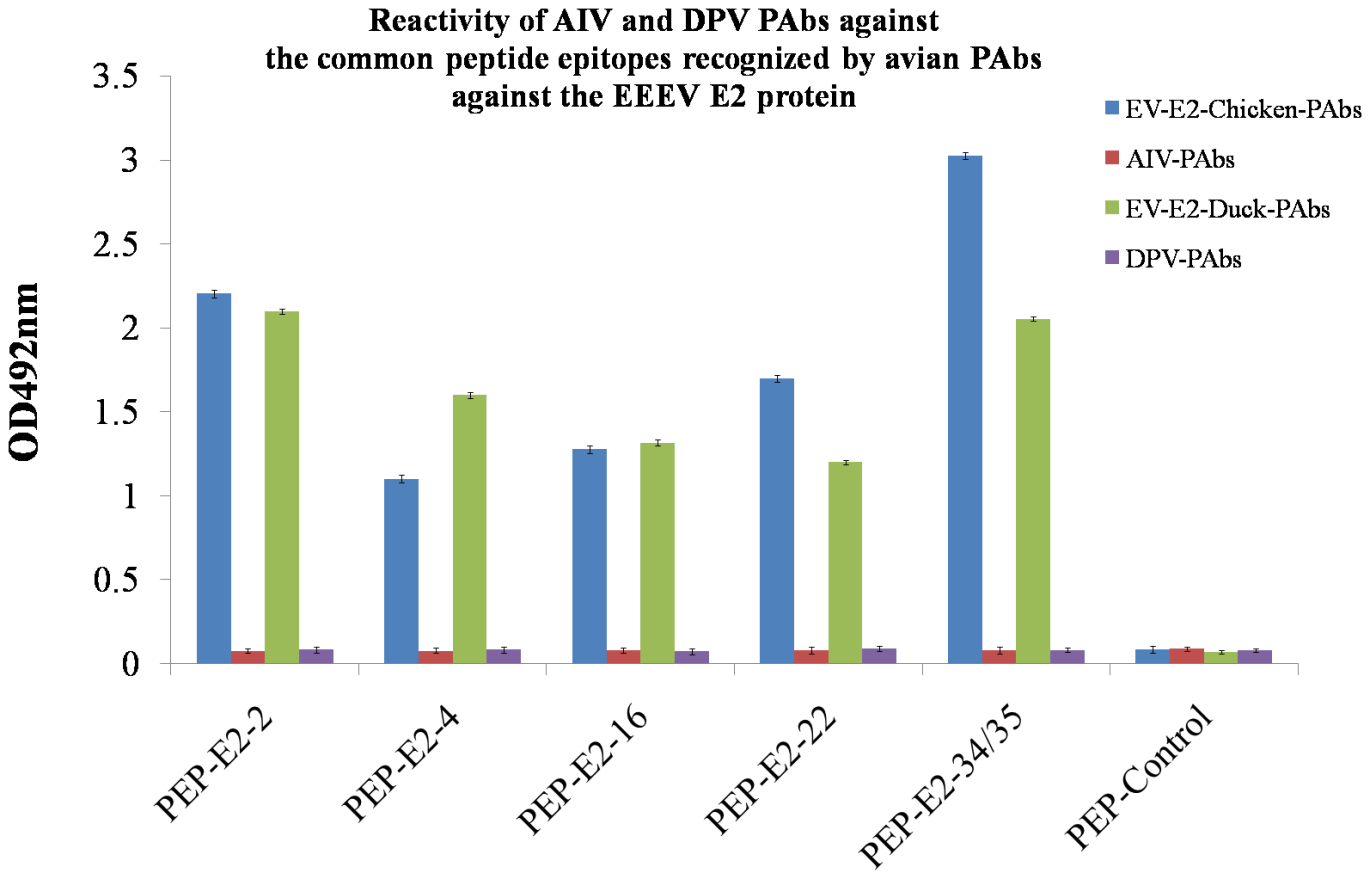
Reactivity of AIV and DPV PAbs against the common epitopes recognized by avian PAbs raised against the EEEV E2 protein. PAbs against AIV and DPV were evaluated for binding to the three common peptide epitopes of EEEV E2 by ELISA. Each bar indicates PAb reactivity as determined by the mean optical density (OD) at 492 nm. Error bars indicate standard deviation. Positive value/Negative value ≥2.1 was considered as positive result.

The five common E2 epitopes were further evaluated using antisera generated in chickens against Avian Influenza Virus (AIV) and in ducks against Duck Plague Virus (DPV). Avian PAbs generated against AIV and DPV did not react with the five E2 epitopes commonly recognized by antisera generated by chickens and ducks with EEEV E2 protein (Figure 5).

## Discussion

In this study, linear peptide epitopes on the EEEV E2 protein were identified using E2-reactive PAbs generated in chickens and ducks. Avian E2-reactive PAbs were elicited by immunizing chickens and ducks with purified recombinant E2 protein and peptide epitopes were identified by screening peptides derived from the amino acid sequence of E2 with the high titer avian PAbs. Twelve peptide epitopes were recognized by chicken PAbs, while thirteen epitopes were recognized by duck PAbs. Six epitopes were recognized by PAbs obtained from both chicken and duck (E2-2/4/16/22/34/35). The peptide E2-34 and 35 were adjacent, we conjectured that the epitope contained may focused on the common region, but the value (OD492nm) against E2-34 was significantly higher than E2-35 (P<0.05), so we used the peptides based E2-34 for further specific identification.

Sequence alignments were performed to define the regions of associated alphaviruses corresponding to the identified common EEEV E2 linear epitopes. Peptides corresponding to the epitope region were synthesized and used as target in an ELISA to evaluate the specificity of the E2 epitope among related alphaviruses. Additionally, PAbs raised against other avian viruses were used as an additional specificity control. Two EEEV antigenic complex-specific epitope and three EEEV subtype I-specific (NA EEEV-specific) epitopes were defined from this analysis. Based on the sequence alignment and the reactivity of PAbs in Figure 3 a, we can infer that ^25^S is a key amino acid in the epitope located at amino acids 11–26 of the EEEV E2 protein. Similarly, we can conclude that ^35^E and ^44^V in E2-4 and ^152^R in E2-16 are essential residues in the corresponding epitopes of the EEEV E2 protein (Figure 3 b and c).

According to results of previous studies, the amino acid 120 (contained in E2-12), 193 (contained in E2-19), 213 (contained in E2-22), 216 (contained in E2-22) and 218 (contained in E2-22) are located at the surface of E2 protein [27], [28]. Amino acids 180–220 (SINV; epitopes E2-18, 19 and 22) probably located on the virus surface and E2-216 (RRV) may contribute to a neutralizing epitope [29]. The amino acid residues locating on 182–207 (E2-19) and 115–119 (E2-12) of VEEV E2 protein are located at the tip of the spikes [30], [31]. And amino acids locating in 55 (E2-6), 116, 117, 116–119 and 120 (E2-11+12) are important for virus receptor binding suggested by mutagenesis and antibody epitope mapping studies [32]. Even though the accurate positions of the epitopes which identified in our work in the E1–E2 heterodimer also need further study.

Emerging infectious zoonoses are of high importance to both human and animal public health. Interest has arisen in using animal health data to inform human public health surveillance activities in many regions. In 1978, the Florida Sentinel Chicken Arboviral Surveillance Program was established to monitor sentinel chickens to detect arboviral activity throughout the state [33]. According to that sentinel chickens may also be the better indicator of EEEV activity, although they were not useful for detecting EEEV activity in all regions [34]. Nonetheless, domestic chickens remain the most widely used sentinel animal NA EEEV, primarily because the time and place of virus exposure can be defined for domestic chicken sentinels. In contrast, the hamster was recognized as the sentinel animal for SA EEEV, especially for Venezuelan isolates. However, the enzootic transmission cycles of SA EEEV are poorly understood and the role of birds versus small mammals as enzootic hosts remains unclear [1]. The avian-specific E2 epitopes and the common immunodominant E2 epitopes identified in our study may offer a mechanism to define the reservoirs and hosts of NA and SA EEEV through an evaluation of the seroprevalence of antibodies against these defined epitopes in different avian species. In our study, the results get from the expressed protein immunized poultry and not the live virus infected animal is a deficiency, so the actual value of applications of the epitopes in diagnosis and vaccine development needs further identification.

## Materials and Methods

### Ethics statement

All animal studies were approved by the Review Board of the Harbin Veterinary Research Institute, Chinese Academy of Agricultural Sciences. The Animal Ethics Committee approval number was Heilongjiang-SYXK 2006-032.

### Avian species, proteins and plasmid

EEEV-negative chickens and ducks were supplied by the Centre of Experimental Animals, Harbin Veterinary Research Institute, Chinese Academy of Agricultural Sciences (CAAS). A set of 42 partially overlapping polypeptides, each 16 amino acids in length, covering the entirety of the EEEV E2 protein (E2-1 to E2-42, Table S1) were generated in our laboratory according to the methods previously described [35], [36]. The E2 sequence used for the experiments in this study was synthesized from an EEEV NA variant strain (GenBank accession number X63135.1) and cloned into pCI-neo, and maintained in our laboratory. The plasmid pShuttle-E2 were constructed and maintained in our laboratory [35], [36].

### Express and purify recombinant EEEV E2 protein

Express and purify recombinant EEEV E2 protein was prepared as previously described [31]. In brief, the E2 gene was cloned into the pFastBac™ vector. The recombinant pFastBac™ vector was then transformed into competent DH10Bac™ *E. coli* cells, and got the colonies containing the recombinant bacmid DNA which appeared white. Insect cells were transfected with recombinant Bacmid DNA by using Cellfectin®. Recombinant protein was analyzed by sodium dodecyl sulfate-polyacrylamide gel electrophoresis (SDS-PAGE) and purified by Ni-nitrilotriacetic acid affinity chromatography (Qiagen) according to the manufacturer's instructions, then identified by WB [35], [36].

### Preparation and characterization of avian PAbs

Five six-week-old chickens and ducks were immunized intradermally and subcutaneously with purified recombinant E2 protein in Freund's complete adjuvant (Sigma, USA), respectively. Animals were administrated two booster immunizations containing purified E2 protein in Freund's incomplete adjuvant at 2-week intervals. Immediately prior to each immunization, blood was collected to measure E2-reactive antibody titers by indirect ELISA and IFA. Two weeks after the final booster immunization, sera were collected and used to define antibody binding epitopes in the EEEV E2 protein.

For indirect ELISAs, purified recombinant E2 protein was plated at 100 ng ml^−1^ as target antigen, the sera from immunized and unimmunized chickens and ducks served as a primary antibody source and were tested at serial ten-fold dilutions (1∶10 to 1∶10^6^). HRP-conjugated goat anti-chicken and rabbit anti-duck secondary antibodies at a 1∶2,000 and 1∶1000 dilutions, respectively, were used in the indirect ELISA. IFA was performed using Sf9 insect cells infected with the E2-expressing recombinant baculovirus BACV-E2, and BHK-21 cells transfected with the E2-expressing eukaryotic expression plasmid pShuttle-E2. Serial two-fold dilutions of sera (1∶2 to 1∶1024) were used for detection. FITC-conjugated goat anti-chicken and rabbit anti-duck secondary antibodies were at a 1∶100 and 1∶50 dilutions, respectively, for the IFA. All the detection repeated three times.

### Comprehensive mapping of epitopes on EEEV E2 protein using avian PAbs by WB

A set of 42 partially overlapping 16-mer peptides obtained from the amino acid sequence of the EEEV E2 protein were expressed as MBP-fused polypeptides. The adjacent peptides had 6 amino acids in common. The screen of antisera against the MBP fusion polypeptides by WB has been described previously [36]. The full-length recombinant E2 protein was used as a positive control, with the MBP-tag serving as a negative control. The sera of immunized or unimmunized poultry at a 1∶100 dilution were used as the primary antibody source. HRP-conjugated goat anti-chicken and rabbit anti-duck secondary antibodies at a 1∶1,000 and 1∶500 dilutions, respectively, were used for detection.

### Further confirmation of the epitopes identified by WB using synthesized peptide ELISA

The polypeptides recognized by avian PAbs by WB were synthesized and used as coating antigen to confirm antibody binding epitopes in the E2 protein (Table 3, Shanghai Bootech Bio Science & Technology, China). The ELISA was performed as described previously [36], [37]. The irrelevant polypeptide (V5-Tag, GKPIPNPLLGLDST) was used as an irrelevant peptide control and the anti-MBP-monoclonal antibody (mAb) was used as an irrelevant antibody control. Sera from unimmunized chickens and ducks served as negative controls. All the sera were used at a 1∶100 dilution, with HRP-conjugated goat anti-chicken and rabbit anti-duck secondary antibodies at a 1∶2,000 and 1∶1000 dilutions, respectively, for detection.

**Table 3:** Synthesized polypeptides used to further identify linear peptide epitopes recognized by WB screening with avian PAbs.

Peptides Denomination	Peptides Sequence
PEP-E2-1	DLDTHFTQYKLARPYI
PEP-E2-2	LARPYIADCPNCGHSR
PEP-E2-3	NCGHSRCDSPIAIEEV
PEP-E2-4	PIAIEEVRGDAHAGVI
PEP-E2-5	HAGVIRIQTSAMFGLK
PEP-E2-6	AMFGLKTDGVDLAYMS
PEP-E2-11	AQCPPGDTVTVGFHDG
PEP-E2-12	VGFHDGPNRHTCTVAH
PEP-E2-15	PPEHGVELPCNRYTHK
PEP-E2-16	NRYTHKRADQGHYVEM
PEP-E2-18	LVADHSLLSIHSAKVK
PEP-E2-19	HSAKVKITVPSGAQVK
PEP-E2-22	SDYTTTCTDVKQCRAY
PEP-E2-24	KKWVYNSGRLPRGEGD
PEP-E2-30	LLTTRSLGSDANPTRQ
PEP-E2-31	ANPTRQWIERPTTVNF
PEP-E2-32	PTTVNFTVTGEGLEYT
PEP-E2-34	PPKRVWAQESGEGNPH
PEP-E2-35	GEGNPHGWPHEVVVYY

### Reactivity of EEEV E2-reactive PAbs with the polypeptides corresponding to the common E2 epitope regions of other alphaviruses

Amino acid alignments were carried out to identify the corresponding regions of E2 protein in related alphaviruses, including EEEV antigen complex (lineage I to IV), VEEV, WEEV, RRV and SINV (Lasergene, DNASTAR Inc., Madison, WI). Representative strains of different alphaviruses were chosen for alignment. The accession numbers of EEEV antigen complex (Lineage I to IV) were X63135 and U01556, AF159559, GU001934 and AF159561, respectively. The accession numbers of VEEV, WEEV, RRV and SINV were ACV42439, ACN87270, AEC49788 and AAC83379, respectively. Based on the results of amino acid alignments, polypeptides that corresponded to the common immunodominant epitopes in alphaviruses were synthesized (Table 4, Shanghai Bootech BioScience&Technology, China). The reactivity of synthesized polypeptides with avian PAbs was evaluated by ELISA as described above. V5-Tag served as an irrelevant polypeptide control, and sera from unimmunized poultry served as negative controls for all peptide ELISAs.

**Table 4:** Synthesized polypeptides used to assess the specificity of five common epitopes.

Peptides Denomination	Peptides Sequence
PEP2-EVNA-I	LARPYIADCPNCGHSR
PEP2-EVSA-II/III	LARPYIADCSNCGHGR
PEP2-EVSA-IV	LARPYIADCPNCGHGR
PEP2-VEEV	PYMAKCVRCAVGS
PEP2-WEEV	PYLGFCPYCRHST
PEP4-EVNA-I	PIAIEEVRGDAHAGVI
PEP4-EVSA-II	PIAIEDIRGDAHAGYI
PEP4-EVSA-III/IV	PIAIEDVRGDAHAGYI
PEP4-VEEV	PIAIEAVRSDGHDGYI
PEP4-WEEV	PIKIEVWDESDDGSI
PEP16-EVNA-I	NRYTHKRADQGHYVEM
PEP16-EVSA-II	TKYTHKRADQGHYVEM
PEP16-EVSA-III/IV	NKYTHKRADQGHYVEM
PEP16-VEEV	RVYVHDAQKKDAYVEM
PEP16-WEEV	HVYDHLKETSAGYITM
PEP22-EVNA-I(1)	SDYTTTCTDVKQCRAY
PEP22-EVNA-I(2)	SDHTTTCTDVKQCRAY
PEP22- EVSA-II	SDYTTACTNLKQCRAY
PEP22- EVSA-III	GDYTTTCTDLKQCRAY
PEP22- EVSA-IV	GEHTTTCTDVKQCRAY
PEP34/35-EEEV-I/III/IV	PPKRVWAQESGEGNPHGWPHEV
PEP34/35-EVSA-II	PPKRVWAQESGEGNPHGWPHEI
PEP34/35-VEEV	DPVRVYAQESAPGDPHGWPHEI
PEP34/35-WEEV	EPVRVWAQESAPGDPHGWPHEI

### Specificity evaluation of the common immunodominant epitopes used other avian virus' antisera

We used antisera generated against AIV and DPV to verify that other avian viruses did not elicit antibody responses against the common EEEV E2 immunodominant epitopes. Five antisera generated against AIV and three antisera generated against DPV were tested for reactivity with the three E2 peptide epitopes recognized commonly by chicken and duck antisera following immunization with EEEV E2 protein by peptide ELISA as described above. The reactivity of synthesized polypeptides with avian PAbs was evaluated by ELISA as described above.

### Location analysis of epitopes in the EEEV E2 protein

Location analysis of epitopes was made on one E2 protein (Accession NO. X63135.1) of EEEV to explain the general spatial relationship using UCSF Chimera 1.7rc according the crystal structure of VEEV E2 protein (PBD accession no. 3J0C-chain K) [38].

### Statistical analysis

ELISA antibody titers were statistically analyzed. Student's *t* tests were used to compare differences in antibody titers between two groups. Statistical significance was defined as *P*<0.05 [39].

## Supporting Information

Table S1 **The complementary oligonucleotide pairs encoding 42 overlapping, 16-mer peptides that cover the entire E2 protein amino acid sequence from an EEEV NA variant strain (GenBank accession number X63135.1).**
(DOC)
