# Supplementary material for: Comprehensive Mapping of Common Immunodominant Epitopes in the Eastern Equine Encephalitis Virus E2 Protein Recognized by Avian Antibody Responses
Source: PLoS One. 2013 Jul 26;8(7):e69349. doi: 10.1371/journal.pone.0069349 (PMC3724843; doi:10.1371/journal.pone.0069349)
Supplement: Table S1 — The complementary oligonucleotide pairs encoding 42 overlapping, 16-mer peptides that cover the entire E2 protein amino acid sequence from an EEEV NA variant strain (GenBank accession number X63135.1). (DOC) [file pone.0069349.s001.doc]

| Table S1  The complementary oligonucleotide pairs encoding 42 overlapping, 16-mer peptides that encompassed the entire E2 amino acid sequence of EEEV | | |
| --- | --- | --- |
| Designations of | The sequences of oligonucleotides | Coding motifs |
| oligonucleotides |  | (designations) |
| EEEV E-1-F | 5'-AATTCgatttggacactcatttcacccagtataagctggcacgcccgtatattTAAG-3' | DLDTHFTQYKLARPYI |
| EEEV E-1-R | 5'-TCGACTTAaatatacgggcgtgccagcttatactgggtgaaatgagtgtccaaatcG-3' |  |
| EEEV E-2-F | 5'-AATTCctggcacgcccgtatattgctgattgccctaactgtgggcatagtcggTAAG-3' | LARPYIADCPNCGHSR |
| EEEV E-2-R | 5'-TCGACTTAccgactatgcccacagttagggcaatcagcaatatacgggcgtgccagG-3' |  |
| EEEV E-3-F | 5'-AATTCaactgtgggcatagtcggtgcgacagccctatagctatagaagaagtcTAAG-3' | NCGHSRCDSPIAIEEV |
| EEEV E-3-R | 5'-TCGACTTAgacttcttctatagctatagggctgtcgcaccgactatgcccacagttG-3' |  |
| EEEV E-4-F | 5'-AATTCgctatagaagaagtcagaggggatgcgcacgcaggagtcatccgcTAAG-3' | IAIEEVRGDAHAGVIR |
| EEEV E-4-R | 5'-TCGACTTAgcggatgactcctgcgtgcgcatcccctctgacttcttctatagcG-3' |  |
| EEEV E-5-F | 5'-AATTCcacgcaggagtcatccgcatccagacatcagctatgttcggtctgaagTAAG-3' | HAGVIRIQTSAMFGLK |
| EEEV E-5-R | 5'-TCGACTTActtcagaccgaacatagctgatgtctggatgcggatgactcctgcgtgG-3' |  |
| EEEV E-6-F | 5'-AATTCgctatgttcggtctgaagacggatggagttgatttggcctacatgagtTAAG-3' | AMFGLKTDGVDLAYMS |
| EEEV E-6-R | 5'-TCGACTTAactcatgtaggccaaatcaactccatccgtcttcagaccgaacatagcG-3' |  |
| EEEV E-7-F | 5'-AATTCgatttggcctacatgagtttcatgaacggcaaaacgcagaaatcaataTAAG-3' | DLAYMSFMNGKTQKSI |
| EEEV E-7-R | 5'-TCGACTTAtattgatttctgcgttttgccgttcatgaaactcatgtaggccaaatcG-3' |  |
| EEEV E-8-F | 5'-AATTCaaaacgcagaaatcaataaagatcgacaacctgcatgtgcgcacctcaTAAG-3' | KTQKSIKIDNLHVRTS |
| EEEV E-8-R | 5'-TCGACTTAtgaggtgcgcacatgcaggttgtcgatctttattgatttctgcgttttG-3' |  |
| EEEV E-9-F | 5'-AATTCctgcatgtgcgcacctcagccccttgttccctcgtgtcgcaccacggcTAAG-3' | LHVRTSAPCSLVSHHG |
| EEEV E-9-R | 5'-TCGACTTAgccgtggtgcgacacgagggaacaaggggctgaggtgcgcacatgcagG-3' |  |
| EEEV E-10-F | 5'-AATTCctcgtgtcgcaccacggctattacatcctggctcaatgcccaccagggTAAG-3' | LVSHHGYYILAQCPPG |
| EEEV E-10-R | 5'-TCGACTTAccctggtgggcattgagccaggatgtaatagccgtggtgcgacacgagG-3' |  |
| EEEV E-11-F | 5'-AATTCgctcaatgcccaccaggggacacggttacagttgggtttcacgacgggTAAG-3' | AQCPPGDTVTVGFHDG |
| EEEV E-11-R | 5'-TCGACTTAcccgtcgtgaaacccaactgtaaccgtgtcccctggtgggcattgagcG-3' |  |
| EEEV E-12-F | 5'-AATTCgttgggtttcacgacgggcctaaccgccatacgtgcacagttgcccatTAAG-3' | VGFHDGPNRHTCTVAH |
| EEEV E-12-R | 5'-TCGACTTAatgggcaactgtgcacgtatggcggttaggcccgtcgtgaaacccaacG-3' |  |
| EEEV E-13-F | 5'-AATTCacgtgcacagttgcccataaggtagaattcaggccagtgggtagagagTAAG-3' | TCTVAHKVEFRPVGRE |
| EEEV E-13-R | 5'-TCGACTTActctctacccactggcctgaattctaccttatgggcaactgtgcacgtG-3' |  |
| EEEV E-14-F | 5'-AATTCaggccagtgggtagagagaaataccgtcacccacctgaacatggagttTAAG-3' | RPVGREKYRHPPEHGV |
| EEEV E-14-R | 5'-TCGACTTAaactccatgttcaggtgggtgacggtatttctctctacccactggcctG-3' |  |
| EEEV E-15-F | 5'-AATTCccacctgaacatggagttgaattaccatgcaaccgttacacccacaagTAAG-3' | PPEHGVELPCNRYTHK |
| EEEV E-15-R | 5'-TCGACTTActtgtgggtgtaacggttgcatggtaattcaactccatgttcaggtggG-3' |  |
| EEEV E-16-F | 5'-AATTCaaccgttacacccacaagcgtgcagaccaaggacactacgttgagatgTAAG-3' | NRYTHKRADQGHYVEM |
| EEEV E-16-R | 5'-TCGACTTAcatctcaacgtagtgtccttggtctgcacgcttgtgggtgtaacggttG-3' |  |
| EEEV E-17-F | 5'-AATTCggacactacgttgagatgcatcaacccgggctagttgccgaccactctTAAG-3' | GHYVEMHQPGLVADHS |
| EEEV E-17-R | 5'-TCGACTTAagagtggtcggcaactagcccgggttgatgcatctcaacgtagtgtccG-3' |  |
| EEEV E-18-F | 5'-AATTCctagttgccgaccactctctccttagcatccacagtgccaaggtgaaaTAAG-3' | LVADHSLLSIHSAKVK |
| EEEV E-18-R | 5'-TCGACTTAtttcaccttggcactgtggatgctaaggagagagtggtcggcaactagG-3' |  |
| EEEV E-19-F | 5'-AATTCcacagtgccaaggtgaaaattacggtaccgagcggcgcccaagtgaaaTAAG-3' | HSAKVKITVPSGAQVK |
| EEEV E-19-R | 5'-TCGACTTAtttcacttgggcgccgctcggtaccgtaattttcaccttggcactgtgG-3' |  |
| EEEV E-20-F | 5'-AATTCagcggcgcccaagtgaaatactactgcaagtgcccagacgtacgagagTAAG-3' | SGAQVKYYCKCPDVRE |
| EEEV E-20-R | 5'-TCGACTTActctcgtacgtctgggcacttgcagtagtatttcacttgggcgccgctG-3' |  |
| EEEV E-21-F | 5'-AATTCtgcccagacgtacgagagggaactaccagcagcgactatacaaccaccTAAG-3' | CPDVREGTTSSDYTTT |
| EEEV E-21-R | 5'-TCGACTTAggtggttgtatagtcgctgctggtagttccctctcgtacgtctgggcaG-3' |  |
| EEEV E-22-F | 5'-AATTCagcgactatacaaccacctgcacggatgtcaaacaatgcagggcttacTAAG-3' | SDYTTTCTDVKQCRAY |
| EEEV E-22-R | 5'-TCGACTTAgtaagccctgcattgtttgacatccgtgcaggtggttgtatagtcgctG-3' |  |
| EEEV E-23-F | 5'-AATTCaaacaatgcagggcttacctgattgacaacaaaaaatgggtgtacaacTAAG-3' | KQCRAYLIDNKKWVYN |
| EEEV E-23-R | 5'-TCGACTTAgttgtacacccattttttgttgtcaatcaggtaagccctgcattgtttG-3' |  |
| EEEV E-24-F | 5'-AATTCaaaaaatgggtgtacaactctggaagactgcctcgaggagagggcgacTAAG-3' | KKWVYNSGRLPRGEGD |
| EEEV E-24-R | 5'-TCGACTTAgtcgccctctcctcgaggcagtcttccagagttgtacacccattttttG-3' |  |
| EEEV E-25-F | 5'-AATTCcctcgaggagagggcgacacttttaaaggaaaacttcatgtgccctttTAAG-3' | PRGEGDTFKGKLHVPF |
| EEEV E-25-R | 5'-TCGACTTAaaagggcacatgaagttttcctttaaaagtgtcgccctctcctcgaggG-3' |  |
| EEEV E-26-F | 5'-AATTCaaacttcatgtgccctttgtgcctgttaaggccaagtgcatcgccacgTAAG-3' | KLHVPFVPVKAKCIAT |
| EEEV E-26-R | 5'-TCGACTTAcgtggcgatgcacttggccttaacaggcacaaagggcacatgaagtttG-3' |  |
| EEEV E-27-F | 5'-AATTCgccaagtgcatcgccacgctggcaccagagcctctagttgagcacaaaTAAG-3' | AKCIATLAPEPLVEHK |
| EEEV E-27-R | 5'-TCGACTTAtttgtgctcaactagaggctctggtgccagcgtggcgatgcacttggcG-3' |  |
| EEEV E-28-F | 5'-AATTCcctctagttgagcacaaacaccgcaccctgattttacacctgtacccgTAAG-3' | PLVEHKHRTLILHLYP |
| EEEV E-28-R | 5'-TCGACTTAcgggtacaggtgtaaaatcagggtgcggtgtttgtgctcaactagaggG-3' |  |
| EEEV E-29-F | 5'-AATTCattttacacctgtacccggaccacccgaccttgctgacgaccaggtcaTAAG-3' | ILHLYPDHPTLLTTRS |
| EEEV E-29-R | 5'-TCGACTTAtgacctggtcgtcagcaaggtcgggtggtccgggtacaggtgtaaaatG-3' |  |
| EEEV E-30-F | 5'-AATTCttgctgacgaccaggtcacttggaagtgatgcaaatccaactcgacaaTAAG-3' | LLTTRSLGSDANPTRQ |
| EEEV E-30-R | 5'-TCGACTTAttgtcgagttggatttgcatcacttccaagtgacctggtcgtcagcaaG-3' |  |
| EEEV E-31-F | 5'-AATTCgcaaatccaactcgacaatggattgagcgaccaacaactgtcaatttcTAAG-3' | ANPTRQWIERPTTVNF |
| EEEV E-31-R | 5'-TCGACTTAgaaattgacagttgttggtcgctcaatccattgtcgagttggatttgcG-3' |  |
| EEEV E-32-F | 5'-AATTCccaacaactgtcaatttcacagtcaccggagaagggttggagtataccTAAG-3' | PTTVNFTVTGEGLEYT |
| EEEV E-32-R | 5'-TCGACTTAggtatactccaacccttctccggtgactgtgaaattgacagttgttggG-3' |  |
| EEEV E-33-F | 5'-AATTCgaagggttggagtatacctggggaaaccatccaccaaaaagagtatggTAAG-3' | EGLEYTWGNHPPKRVW |
| EEEV E-33-R | 5'-TCGACTTAccatactctttttggtggatggtttccccaggtatactccaacccttcG-3' |  |
| EEEV E-34-F | 5'-AATTCccaccaaaaagagtatgggctcaagagtcaggagaagggaatccacatTAAG-3' | PPKRVWAQESGEGNPH |
| EEEV E-34-R | 5'-TCGACTTAatgtggattcccttctcctgactcttgagcccatactctttttggtggG-3' |  |
| EEEV E-35-F | 5'-AATTCggagaagggaatccacatggatggccgcacgaagtggtagtctattacTAAG-3' | GEGNPHGWPHEVVVYY |
| EEEV E-35-R | 5'-TCGACTTAgtaatagactaccacttcgtgcggccatccatgtggattcccttctccG-3' |  |
| EEEV E-36-F | 5'-AATTCgaagtggtagtctattactacaacagatacccattaaccacaattatcTAAG-3' | EVVVYYYNRYPLTTII |
| EEEV E-36-R | 5'-TCGACTTAgataattgtggttaatgggtatctgttgtagtaatagactaccacttcG-3' |  |
| EEEV E-37-F | 5'-AATTCccattaaccacaattatcgggttatgcacctgtgtggctatcatcatgTAAG-3' | PLTTIIGLCTCVAIIM |
| EEEV E-37-R | 5'-TCGACTTAcatgatgatagccacacaggtgcataacccgataattgtggttaatggG-3' |  |
| EEEV E-38-F | 5'-AATTCtgtgtggctatcatcatggtctcttgtgtcacatccgtgtggctccttTAAG-3' | CVAIIMVSCVTSVWLL |
| EEEV E-38-R | 5'-TCGACTTAaaggagccacacggatgtgacacaagagaccatgatgatagccacacaG-3' |  |
| EEEV E-39-F | 5'-AATTCacatccgtgtggctcctttgcaggactcgcaatctttgcataaccccgTAAG-3' | TSVWLLCRTRNLCITP |
| EEEV E-39-R | 5'-TCGACTTAcggggttatgcaaagattgcgagtcctgcaaaggagccacacggatgtG-3' |  |
| EEEV E-40-F | 5'-AATTCaatctttgcataaccccgtataaactagccccgaacgctcaagtcccaTAAG-3' | NLCITPYKLAPNAQVP |
| EEEV E-40-R | 5'-TCGACTTAtgggacttgagcgttcggggctagtttatacggggttatgcaaagattG-3' |  |
| EEEV E-41-F | 5'-AATTCccgaacgctcaagtcccaatactcctggcgttactttgctgcattaagTAAG-3' | PNAQVPILLALLCCIK |
| EEEV E-41-R | 5'-TCGACTTActtaatgcagcaaagtaacgccaggagtattgggacttgagcgttcggG-3' |  |
| EEEV E-42-F | 5'-AATTCttactttgctgcattaagccgacgagggcaTAAG-3' | LLCCIKPTRA |
| EEEV E-42-R | 5'-TCGACTTAtgccctcgtcggcttaatgcagcaaagtaaG-3' |  |
